# Follow-up in healthy schoolchildren and in adolescents with DOWN syndrome: psycho-environmental and genetic determinants of physical activity and its impact on fitness, cardiovascular diseases, inflammatory biomarkers and mental health; the UP&DOWN Study

**DOI:** 10.1186/1471-2458-14-400

**Published:** 2014-04-25

**Authors:** José Castro-Piñero, Ana Carbonell-Baeza, David Martinez-Gomez, Sonia Gómez-Martínez, Verónica Cabanas-Sánchez, Catalina Santiago, Ana M Veses, Fernando Bandrés, Ana Gonzalez-Galo, Félix Gomez-Gallego, Oscar L Veiga, Jonatan R Ruiz, Ascensión Marcos

**Affiliations:** 1 Department of Physical Education, School of Education, University of Cádiz, Puerto Real, Spain; 2 Department of Physical Education, Sports and Human Movement, Faculty of Teacher Training and Education, Autonomous University of Madrid, Madrid, Spain; 3 Immunonutrition Research Group. Department of Metabolism and Nutrition. Institute of Food Science and Technology and Nutrition (ICTAN), Spanish National Research Council (CSIC), Madrid, Spain; 4 School of Doctoral Studies & Research, European University, Madrid, Spain; 5 Department of Toxicology and Health Sanitary, Complutense University of Madrid, Madrid, Spain; 6 PROFITH “PROmoting FITness and Health through physical activity” research group, Department of Physical Education and Sport, Faculty of Sport Sciences, University of Granada, Granada, Spain

## Abstract

**Background:**

An objective diagnosis of sedentary behaviour as well as of the physical activity and fitness levels in youth and to better understand how lifestyle is associated with cardiovascular disease risk factors and other phenotypes is of clinical and public health interest, and might be informative for developing intervention studies focused on the promotion of physical activity in these population. The aim of this methodological paper is to describe the design and assessment in the UP&DOWN study.

**Methods/Design:**

The UP&DOWN study is a multi-center follow-up design where 2225 Spanish primary and secondary schoolchildren from Cadiz and Madrid, respectively, as well as 110 Spanish adolescents with Down syndrome from Madrid and Toledo were recruited to be assessed. Nine main measurement categories are assessed: i) socio-demographic and early determinants; ii) environmental determinants; iii) physical activity and sedentary behaviour; iv) health-related fitness; v) blood pressure and resting heart rate; vi) mental health; vii) dietary patterns; viii) blood samples; and ix) genetic analysis. During the 3-yr follow-up study, socio-demographic and early determinants, and genetic analysis are only assessed in the first year. Blood sampling is assessed in the first year and the third year (2nd follow-up), and all the other measurements are assessed every year.

**Discussion:**

The findings of the UP&DOWN study may help the Health Information Systems and policy makers to identify the target population for primary prevention and health promotion policies, and to develop and test preventive strategies. Moreover, these data will allow following the trends at population level, as well as to modify/adapt/create new evidence-based physical activity guidelines at national level. The findings will also serve as a scientific platform for interventional studies.

## Background

Cardiovascular disease is the leading cause of global mortality [1]. Cardiovascular disease events occur most frequently during or after the fifth decade of life, however, there is evidence indicating that the precursors of cardiovascular disease have their origin in childhood and adolescence [2,3]. Adverse cardiovascular disease risk factors during childhood have been shown to track into adulthood [4]. Therefore, a greater in-depth knowledge of the factors affecting cardiovascular disease risk factors in youth will contribute to the development of effective prevention programs, counselling and public health policy. The protective effect of physical activity (PA) as well as fitness on cardiovascular disease risk and mortality has been reported in people of all ages [5,6].

The determinants of early risk factors for cardiovascular disease, how they change over time, and how changes in lifestyle factors affect the risk of cardiovascular disease in childhood and adolescence is not well understood. There are reasons to believe that the genotype-environment interactions could also be involved in the susceptibility of individuals to develop early risk factors for cardiovascular disease such as insulin resistance, hypertension, dyslipidemia, obesity, and chronic inflammation.

Definition of these interaction effects for phenotypes related to these diseases is therefore important because it will eventually allow the identification of individuals at risk of the development of complications and the identification of those likely to be resistant to lifestyle interventions. The study of these genetic markers in children and adolescents and their relationship to several phenotypic characteristics of the population will permit a better understanding of the pathogenic mechanisms that are involved in non-communicable diseases, specifically, cardiovascular disease and diabetes.

Down syndrome (DS) is associated with a trisomy in chromosome 21. The prevalence of DS is one out of 700–1000 live births [7,8]. Fortunately, life expectancy of DS has increased from ≈ 9 years of age in 1929 [9] to ≈ 55 years of age nowadays [7,8]. DS suffer from many concurrent ailments and there is increasing evidence that the most common pathologies such as muscle hypotonicity, hypermobility of the joints or ligamentour laxity, obesity, undeveloped cardiovascular and respiratory system and short stature (short legs and arms in relation to torso) might be modified by increasing levels of PA and fitness [10]. Assessments of PA in adolescent with DS might be a challenge using subjective measures and only several attempts have been conducted with objective measures [11]. Similarly, fitness assessments is also complex in this population [10]. Studies examining the association of PA and fitness with obesity and related risk factors in this population are scarce, and the evidence mainly comes from to cross-sectional studies conducted in relatively small samples.

To have an objective diagnosis of sedentary behaviour as well as of the PA and fitness levels in youth and to better understand how lifestyle is associated with cardiovascular disease risk factors and other phenotypes is of clinical and public health interest, and might be informative for developing intervention studies focused on the promotion of PA in these population.

In the context of a remarkable increase of social awareness about the problem of physical inactivity in the population, in May 2009 the Spanish National Sports Council launched the Integral Plan on Physical Activity and Sport (A + D Plan). The main aim of this Plan was to drive coordinated actions to promote PA and sports in the Spanish population. The plan emphasised the need of objective information in order to develop nation-wide promotion strategies. Moreover, in 2005 the NAOS Strategy (Strategy for Nutrition, Physical Activity and the Prevention of Obesity) was set up by the Ministry of Health and Consumer Affairs, through the Spanish Agency for Food Safety and Nutrition, with the aim of making the population more aware of the problems obesity means for health, and of promoting any initiatives that help to encourage citizens, particularly children and young people, to adopt healthy lifestyles, mainly through healthy diets and regular physical activity. Thus, the UP&DOWN objectives will contribute to this plan and strategy in several aspects: (i) PA and health promotion; (ii) promotion of PA and sports in school population; (iii) promotion of PA and sports in disabled people; (iv) gender issues related to PA and sports; and (v) PA and sports in people at deprived social risk. Therefore, the results obtained from the UP&DOWN study will be valuable for future development of the Spanish A + D Plan, and in addition they will be helpful to produce relevant scientific information and contribute to the NAOS Strategy to inform public opinion about the risks of obesity and physical inactivity for health. The purpose of this paper is to provide an overview on the design, procedures and methods used in the UP&DOWN study.

### Objectives

1. To determine the patterns, interrelationships and impact on health indicators of objectively and subjectively measured PA, sedentary behaviours and health-related fitness (including fatness) in healthy primary and secondary schoolchildren, and in adolescents with DS along a 3-yr follow-up.

2. To identify the main psychosocial and environmental determinants of PA and sedentary behaviours in healthy primary and secondary schoolchildren, and in adolescents with DS along a 3-yr follow-up.

3. To investigate genotype-lifestyle interactions in healthy primary and secondary schoolchildren, and in adolescents with DS along a 3-yr follow-up.

4. To evaluate the association of PA, sedentary behaviours and health-related fitness with traditional and new cardiovascular disease risk factors in healthy primary and secondary schoolchildren, and in adolescents with DS along a 3-yr follow-up.

## Methods/Design

### Study design

The UP&DOWN study is a multi-center longitudinal design endorsed by the National Research & Development & Innovation Plan of the Spanish Ministry of Education and Science, under the Strategic Line of Sport and Physical Activity.

The management of the UP&DOWN study was designed to ensure effective collaboration and communication among the 4 research centers (Spanish National Research Council (CSIC), University of Cadiz (UCA), Autonomous University of Madrid (UAM), and Complutense University of Madrid (UCM)) involved in the longitudinal study. All centers adhered to a common study protocol for training, implementation of fieldwork, data collection and management, and quality control procedures. At least, once a year a consortium meeting is organized to analyze the process and to implement actions resulting from these meetings, where appropriate.

The UP&DOWN study overall coordination is developed by CSIC. The principal investigator of each center manages the day-by-day coordination of their work packages, and assists the coordinator group on decisions that need to be taken within a time frame that does not allow a full meeting of all partners. An UP&DOWN Core Group, composed of 5 members, outlines the scientific strategy, monitors the progress in view of the overall project timeline and key milestones, and assists the principal investigators on an *ad hoc* basis for all decisions that need to be taken within a time frame that does not allow a full meeting of all partners, or in cases when immediate contact with one or more partners is not possible.

### Participants and selection criteria

UP&DOWN participants include apparently healthy Spanish children and adolescents from primary and secondary schools from regions of Cadiz and Madrid, respectively, as well as adolescents with DS centers from regions of Madrid and Toledo. The UP&DOWN study establishes following inclusion criteria:

– Participants’ selection criteria for healthy children and adolescents: i) to study in 1st/4th grades (6–7 and 9–10 years old, respectively) for children, and 7th/10th grades (12–13 and 15–16 years old, respectively) for adolescents at baseline; ii) do not have physical disability or health problems, which might limit levels of PA.

– Participants’ selection criteria for adolescents with DS: i) to be 11–20 years old with DS; ii) To have an intelligence quotient over 35; iii) do not have physical disability for doing PA.

**Figure 1:**
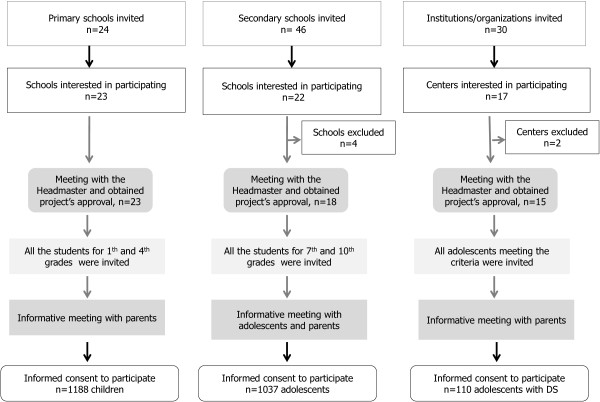
Flow diagram of the study participants.

Figure 1 illustrates the participant flow during the recruitment process. An invitation letter to participate in this study was sent to the headmasters or physical education teachers in each school. A total of 23 primary school, 22 secondary schools and 18 special education centers, accepted the invitation. Four secondary schools and two special education centers were excluded for logistical reasons (be located at a great distance from the city or to have a lower number of students meeting the inclusion criteria). A meeting with the headmasters was performed to obtain the center’s agreement. Next, all children from 1st and 4th grades, adolescents from 7th and 10th grades and adolescents with DS were invited to participate in the study. The parents of students received a brief flyer describing the study, including inclusion criteria and an invitation to attend an informative meeting at the school. A meeting explaining the study purpose was performed in all the centers and an informed consent for their parents/tutors was collected. Finally, 1188 children, 1037 adolescents and 110 adolescents with DS agreed to participate in the study.

### Outcome measures

During the UP&DOWN study 9 main measurement categories are assessed: 1) socio-demographic and early determinants; 2) environmental determinants; 3) PA and sedentary behaviour; 4) health-related fitness; 5) blood pressure and resting heart rate; 6) mental health; 7) dietary patterns; 8) blood samples; and 9) genetic analysis.

**Figure 2:**
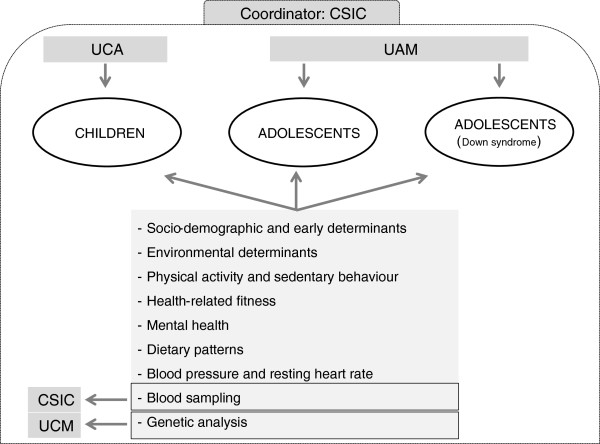
Characteristics on management and data collection in the UP&DOWN study.

All these measurements are assessed in primary schoolchildren from Cadiz (UCA), and secondary schoolchildren and DS adolescents from Madrid and Toledo (UAM) (Figure 2). Blood samples are analyzed in one lab placed at CSIC (Institute of Food Science, and Technology and Nutrition), except for haemogram that is analysed by the same methodologies in two external labs (Cadiz and Madrid) due to the short spanlife blood cells (Figure 2). The same methodologies are used in both labs. Saliva samples are analyzed in Biomedicine laboratory at European University of Madrid in collaboration with the UCM (Figure 2). It should be highlighted that all groups have already collaborated in other co-joint projects (i.e. AVENA, HELENA, AFINOS, EVASYON).

**Figure 3:**
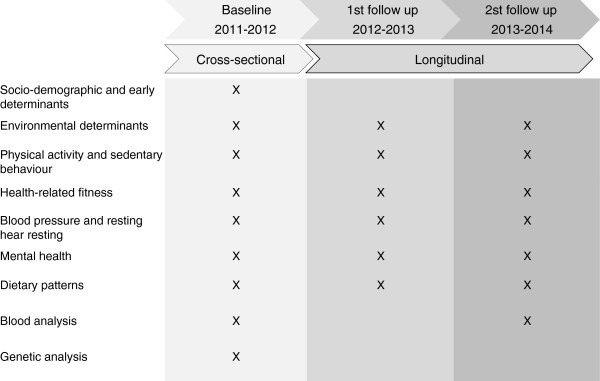
Study assessment schedule.

During the 3-yr follow-up study, socio-demographic and early determinants, and genetic analysis are only assessed in the first year (baseline). Blood sampling is assessed in the first year and the third year (2nd follow-up), and all the other measurements are assessed every year (Figure 3).

#### *1)* Socio-demographic and early determinants

Parents or guardians complete a ‘family questionnaire’, which is a compendium of several questionnaires. Information about socio-demographic factors (e.g. sex, age, birth date, place of birth, time residing in Spain, family structure) is assessed by standardized questions used in previous studies [12,13]. Socioeconomic status is assessed by the Family Affluence Scale [14].

Additionally, parents complete a questionnaire that will address their educational level and occupation status, weight, height, and age, as well as their habitual PA and sedentary behaviours by the short version of the International PA Questionnaire [15]. The family questionnaire also includes questions on several early life health determinants (e.g. child birth weight and height, week of gestation, breastfeeding, smoke and PA habits during pregnancy, motor development during the first years of life) [16-18].

#### *2) Environmental determinants*

Environmental determinants are studied in order to identify their possible influence on PA and sedentary behaviours. In this sense, neighbourhood environmental determinants of PA are assessed using an adapted version of the European questionnaire developed in the ALPHA project [19]. Schools environment is evaluated by two instruments based on the School Health Environment Survey from SHAPES Project (http://www.shapes.uwaterloo.ca): (i) an audit performed by the research group and (ii) a questionnaire completed by participants. Both instruments include questions about density and use of sports facilities at the school, extracurricular sports programs and competitions, organized PA and facilities for active commuting (e.g. bicycle parking, promoting active commuting, etc.). Some PA barriers such as parental permissions [20] and accompanied preferences for active commuting to school are also assessed.

#### *3)* PA and sedentary behaviour

The ActiGraph accelerometer models GT1M, GT3X and GT3X + (Actigraph TM, LLC, Fort Walton Beach, FL, USA) are used to obtain detailed and objective information about daily PA and sedentary behaviour over seven consecutive days. Previous studies have demonstrated that there is strong agreement between measures from GT1M, GT3X and GT3X + activity monitors without needing additional calibration [21,22], as well as they are technically reliable instruments [23]. The accelerometer is attached tightly in the hip, on the back side, with the notch faced upwards, and participants are instructed to use the accelerometer during waking hours and remove it during water-based activities; according to established procedures [24]. The epoch length is analyzed to 10 seconds to allow a more detailed estimate of PA intensity. The screening and data processing procedures to estimate sedentary time, total PA, and PA at different intensities are consistent with previous studies in children and adolescents [25-27].

PA is also assessed with the PA Questionnaire for Adolescents/Children (PAQ-A/C) [28]. This is a self-administered, 7-day recall instrument, with 9-items scored in a five-point scale. The PAQ-A/C provides a final composite activity score, by taking the mean of the 9 items. This questionnaire has been shown to be valid and reliable [28,29]. Extracurricular PA is assessed by the Finnish PA Index [30]*.* It consists of 5 items concerning frequency and intensity of extracurricular PA and participation in organized sports. Using these five items an index of PA is calculated.

The time spent in sedentary behaviours is assessed by the Youth Sedentary Behaviour Questionnaire (YSBQ). The YSBQ was designed in the UP&DOWN framework to assess the amount of time spent in 16 behaviours that were proposed previously by Biddle et al. [31]: (i) watching TV/videos, (ii) playing computer/video games, (iii) internet surfing, (iv) doing homework/study without computer, (v) doing homework/study with computer, (vi) reading for fun, (vii) talking on the telephone, (viii) listening to music, (ix) sitting and talking with family and friends, (x) sitting to rest, (xi) hanging out, (xii) playing exercise videogames, (xiii) doing behavioural hobbies, (xiv) doing cognitive hobbies, (xv) travelling on motorized transport, and (xvi) doing sports/exercise. The items are completed separately for weekdays and weekend days, referring to the last week. Response options are none, 30 minutes, 1 hour, 1 hour and a half, 2 hours, 2 hours and a half, 3 hours, 4 hours, and 5 hours or more. Moreover, we created 6 comparative scales regarding how long participants spend (i) watching TV/videos, (ii) playing computer/video games, (iii) internet surfing, (iv) doing homework/study and (v) doing sports/exercise, and (vi) sitting/lying in comparison with their peers with same age and sex. The response categories are: much less than others, some less than others, as well as others, some more than others and more than others.

Information on the time that their parents, siblings and friends spend in PA and sedentary behaviours, as well as the frequency doing PA and sedentary behaviours with them is also obtained by an internally developed self-report. Information on how much sport and electronic equipment children and adolescents have available at home is obtained. PA facilities checklists were designed by reference to some previous instruments [32-34]. Checklist to assess available electronic equipment at children and adolescents homes and rooms was developed by adapting an existing instrument by Rosenberg et al. [35]. The Pros & Cons PACE scale is also used to assess the pros and cons of reducing sedentary behaviours [36]. This questionnaire asks about the importance of each statement when deciding whether or not to engage in a sedentary behaviour, with responses ranging from not important (1) to extremely important (5). Additionally, we developed some questions regarding parent’s restrictions of sedentary behaviours (e.g. TV watching, telephone/mobile, console and computer use) separately for weekdays and weekend days.

#### *4)* Health-related fitness

Health-related fitness is assessed by field-based tests and self-report using the ALPHA health-related fitness test battery [37,38] and the International Fitness Scale (IFIS) [39], respectively.

The extended version of the ALPHA health-related fitness test battery includes the following tests: (i) the 20 m shuttle run test to assess cardiorespiratory fitness; (ii) the handgrip strength and (iii) standing broad jump to assess musculoskeletal fitness, (iv) the 4 × 10 m to assess motor fitness, and (v) body mass index, (vi) waist circumference, and (vii) skinfold thickness (triceps and subscapular) to assess body composition.

(i) 20 m shuttle run test*:* The participants are required to run between two lines 20 m apart, while keeping pace with audio signals emitted from a pre-recorded CD. The initial speed is 8.5 km/h, and is increased by 0.5 km/h per minute (one minute equals one stage). Participants are instructed to run in a straight line, to pivot on completing a shuttle, and to pace themselves in accordance with the audio signals. The test will finish when the participant fails to reach the end lines concurrent with the audio signals on two consecutive occasions, or when the subject stops because of fatigue. The participants are encouraged to keep running as long as possible throughout the course of the test. The test is performed once and the last completed stage or half-stage at which the subject dropped out will be scored. A gymnasium or space large enough to mark out a 20 m track will be used to perform the test.

(ii) Handgrip strength test: To perform this test a hand dynamometer with adjustable grip (TKK 5101 Grip D; Takey, Tokio, Japan) will be used. This dynamometer presents a high validity and reliability when compared with calibrated known weights [40]. The participant squeezes gradually and continuously for at least two seconds, performing the test with the right and left hands in turn, and with the elbow in full extension as described elsewhere [40]. The grip-span of the dynamometer is adjusted according to the hand size for determining the maximum handgrip strength using the equations specifically developed for children [41] and adolescents [42]. The test is performed twice and the maximum score for each hand is recorded in kilograms. The sum of the scores achieved by left and right hands is used in the analysis.

(iii) Standing long jump test: From a starting position immediately behind a line, standing with feet approximately shoulder’s width apart, the participant jumps as far forwards as possible on a non-slip hard surface. The test is performed twice and the best score is recorded in centimetres.

(iv) 4 × 10 m shuttle run test: 4 × 10 m shuttle run is an adaptation of the 5 × 10 m shuttle run test included in the EUROFIT battery [43] but maintaining the same characteristics. Velocity, agility and coordination are assessed in this test. Participant run 4 × 10 m (back and forth) as fast as possible. The test is performed twice and the best score is recorded in seconds: hundredths.

(v) Body mass index: Participant is barefoot and with T-shirt and short trousers. Weight is measured with an electronic scale (Type SECA 861; range, 0.05 to 130 kg; precision, 0.05 kg), and stature is measured in the Frankfort plane with a telescopic stature-measuring instrument (Type SECA 225; range, 60 to 200 cm; precision, 1 mm). Body mass index is calculated as weight/height squared (kg/m^2^). Participant is categorized according to the BMI international cut-off values as underweight, normalweight, overweight, and obesity [44,45]. Weight and stature are carried out twice, but not consecutively, and mean of the two measurements is used in the analyses.

(vi) Waist circumference: Waist circumference is measured with a non-elastic tape (SECA 200; range, 0 to 150 cm; precision, 1 mm), at the level of the natural waist, in a horizontal plane, which is the narrowest part of the torso, as seen from a front view. In some obese participants, it is difficult to identify the waist circumference, therefore this measurement is performed in the midpoint between the superior iliac spine and the costal edge in the midaxillary line [46]. The measurements are taken at the end of a normal expiration, without the tape compressing the skin. The measurements are carried out twice, but not consecutively, and mean of the two measurements is used in the analyses.

(vii) Skinfolds: Triceps and subscapular skinfold thickness are measured on the non-dominant side of the body with a Holtain caliper (range, 0 to 40 mm; precision, 0.2 mm). Triceps is raised in a vertical fold halfway between the acromion process and the superior head of the radius, in the posterior aspect of the arm, and subscapular about 20 mm below the inferior angle of the scapula and 45° to the lateral side of the body. It is performed according to Lohman’s anthropometric standardization reference manual [47]. Triceps and subscapular skinfold thickness are carried out twice, but not consecutively, and mean of the two measurements is used in the analyses.

Additionally, we assess sitting height and neck circumference. Both, has been associated with cardiovascular risk factors in youth [48,49]. Sitting height is assessed with the participant sits on the box with his or her back and buttocks to the backboard of the stadiometer (Type SECA 225; range, 60 to 200 cm; precision, 1 mm), and with his or her head in the Frankfort horizontal plane. The knees are directed straight ahead with the arms and hands resting at the sides [50]. The measurements are carried out twice, but not consecutively, and mean of the two measurements is used in the analyses. Neck circumference is assessed with the participants standing in an erect position, hanging their arms freely and keeping their head aligned in the Frankfort horizontal plane. The superior border of a non-elastic tape measure (SECA 200; range, 0 to 150 cm; precision, 1 mm) is placed just below the laryngeal prominence and applied perpendicular to the long axis of the neck [50]. The measurements are carried out twice, but not consecutively, and mean of the two measurements is used in the analyses.

The recommended sequence to administer this battery would be as follows: (1) pubertal status, (2) weight, height and sitting height (3) waist and neck circumferences, (4) skinfold thickness (triceps and subscapular), (5) handgrip strength, standing long jump and 4 × 10 m shuttle run test, and (6) 20 m shuttle run test. The ALPHA health-related fitness test battery also suggest an evaluation of the pubertal development of children and adolescents in order to classify themselves in one of the five stages of pubertal maturity defined by Tanner and Whitehouse [51].

The IFIS is a simple and short self-administered scale to assess physical fitness. The IFIS is composed of five Likert-scale questions about the perceived youth’ overall fitness and the main components: cardiorrespiratory fitness, muscular fitness, speed-agility, and flexibility in comparison with their friends’ physical fitness (very poor, poor, average, good and very good) [39].

#### *5)* Blood pressure and resting heart rate

Blood pressure is measured by a validated digital automatic blood pressure monitor (OMRON M6, OMRON HEALTH CARE Co., Ltd., Kyoto, Japan) according to the International Protocol of the European Society of Hypertension [52]. Resting heart rate is also recorded using the same blood pressure monitor.

#### *6) Mental health*

Positive and negative affect, subjective health, dispositional hope, health-related quality of life, eating disorders and academic performance are assessed.

Affectivity is assessed by the PANASN scale of positive and negative affects for children and adolescents [53]. The PANASN consists of 20 adjective descriptors of general mood/affect broken down into two 10-item subscales of positive affect and negative affect, respectively. Participants rate each item on a 5-point Likert scale.

Subjective health is assessed by the classic self rated health status item that consists of asking respondents to rate their health as “excellent, good, fair, bad or poor”. The Children´s Hope Scale assess levels of hopeful [54]. This scale is comprised of 6-item that assess pathways thinking (the children’s perceived capability to produce routes to those goals) and agency thinking (perception that children can initiate and sustain action toward a desired goal). Participants rate each item on a 6-point scale. Health-related quality of life is evaluated by the KIDSCREEN-10, which consists of a 10-item scale. Participants rate each item on a 5-point Likert scale [55].

The risk of eating disorders is assessed by the SCOFF questionnaire, which has been validated for Spanish youth [56]*.* It is a screening instrument designed to be routinely used at population level to identify individuals at risk for eating disorders such as anorexia nervosa, bulimia, and binge eating. The questionnaire consists of five eating-related questions asking about intentional vomiting, loss of control over eating, weight loss, body dissatisfaction and food intrusive thoughts. Answering positively two or more items of SCOFF questionnaire has been suggested as the threshold for a suspicion of a probable eating disorder case [57].

Academic performance is assessed through grades reported by every school. Four main indicators are used: (i) Mathematics, (ii) Language, (iii) average of Mathematics and Language, and (iv) grade point average. Additionally, three school factors related to academic performance are also obtained (i.e. school attitude, repeating, absenteeism) [58].

#### *7) Dietary patterns*

Both children and adolescents complete a food preference questionnaire. Information about dietary habits and eating behaviour (i.e. eating breakfast, meals’ frequency per day) is recorded similarly than in previous studies [13,59]. Adherence to the Mediterranean diet is evaluated using the KIDMED index [60].

#### *8) Blood sampling*

A fasting blood sample is obtained from the cubital vein in the early morning at the schools attended by children and adolescents in the subset. At least 14 ml of blood are drawn from each subject and are aliquoted into 3 tubes: 1 tube containing EDTA (2.5 ml), 1 tube sodium citrate (1.5 ml) and 1 tube containing dry gel for serum (10 ml). Once the blood is collected, it is immediately transported to standard laboratories in each city. The anticoagulated blood in EDTA (2.5 ml) are analysed to obtain haemogram data. The remainder of the blood (dried gel and sodium citrate) is centrifuged; serum and plasma are removed and then frozen at -80°C to be analysed later. The haematological, biochemical and cardiometabolic variables measured in the UP&DOWN studies are shown in Table 1.

**Table 1:** Haematological, biochemical and cardiometabolic variables

**Haematological profile**	**Analytical techniques**
Red blood cell, white blood cell, neutrophil, lymphocyte, monocyte, basophil, eosinophil, platelet counts, leukocyte profile, haemoglobin concentrations, haematocrit and haematic indices.	Automatic cell counter
**Biochemical variables**	**Analytical techniques**
Triglycerides, total cholesterol (c), HDL-c, LDL-c, glucose, and total proteins	Colorimetric assay (AU2700 Olympus analyser)
**Cardio-metabolic variables and inflammatory**	**Analytical techniques**
Visfatine	Enzyme-linked immunoSobent assay (Human visfatin Elisa kit; Cusabio Biotech)
TNF-α, IL-6, adiponectin, insulin, leptin	Immunoassay (xMAP Techonology) using a kit (5 + 1) plex: 171B5006M Bio-Plex Human IL-6 set; 171B5026M Bio-Plex Human TNF-α set; 171D50001 Bio-Plex Human Cytokine Stds; 171-A7003M Bio-plex Pro Human Diabetes Adiponectin Assay; YB0000002Y Bio-Plex Human Diabetes 3-Plex Assay
Cortisol	Enzyme-Linked ImmunoSorbent Assay (ARBOR ASSAYS kit)
C3 and C4 complement factors, and CRP	Turbidimetry (AU2700 Olympus analyser)
Fibrinogen	Clauss method
Galectin-3	Enzyme-Linked ImmunoSorbent Assay (Omnikine ^TM^ Human Galectin- 3 Elisa Kit, assay biotech)

HDL-c: high density lipoprotein cholesterol; LDL-c: low density lipoprotein cholesterol; TNF-α: tumor necrosis factor alpha; IL-6: inteleukin 6; C3: complement factor 3; C4: complement factor 4; CRP: complement factor reactive protein.

#### *9) Genetic analysis*

Genomic Deoxyribonucleic acid is extracted from buccal cells collected on swabs or FTA® cards, according to standard phenol/chloroform procedures followed by alcohol precipitation.

Allelic discrimination analysis is performed by pre-designed Life Technologies TaqMan® SNP Genotyping Assays on demand for the fat mass and obesity associated (FTO) rs9939609 polymorphism (ID: C_30090620_10) and for the peroxisome proliferator-activated receptor gamma coactivator 1-alpha (PGC1-α) rs8192678 polymorphism (ID: C_1643192_20).

Polymerase Chain Reaction (PCR) amplification is performed using a StepOne™ Real-Time PCR System (Life Technologies, Foster City, CA) with a denaturation stage at 95°C for 10 min, 50 cycles of denaturation at 92°C for 15 sec, annealing/extension at 60°C for 1 min, and a final extension stage of 30 sec at 60°C.

Allelic discrimination analysis for the angiotensin-converting enzyme (ACE) I/D polymorphism is performed by PCR followed by electrophoresis on a 1.5% agarose gel containing ethidium bromide. The primers used are: 5′-CTGGAGAGCCACTCCCATCCTTTCT and 5′-GACGTGGCCATCACATTCGTCAGAT [61]. The fragments amplified are a 190 product for allele D (allele without insertion) and a 490 bp product for allele I (allele with insertion). In order to avoid a misclassification of ID heterozygotes as DD homozygotes, a second PCR reaction is performed in all samples initially classified as DD with these insertion-specific primer pair: 5′-TGGGACCACAGCGCCCGCCACTAC and 5′-TCGCCAGCCCTCCCATGCCCATAA [62]. Only the allele I produced a 335 bp fragment, identified on a 1.5% agarose gel stained with ethidium bromide performed in all of the samples initially classified as DD with these.

#### Other lifestyles and health indicators

Information on the following lifestyles and health indicators is also obtained by self-report: sleep duration, alcohol and tobacco consumption, morning and during the day tiredness, and time spent with friends per week [63]; back painful [64]; go late to sleep because of watching TV, and time spent at home during week and weekend days.

### Standardization and harmonization

Both during the feasibility study and for the follow-up study, there is a strict standardization of the fieldwork. Methodological coordination and harmonization workshops related to the evaluation of questionnaires, the measurements of anthropometry, PA, fitness, as well as biological sample transportation and conservation are held up in order to carry out the same methods, both in children and adolescents, as well as in adolescents with DS.

To validate and harmonize the methodology, a general training workshop was held in Madrid (Spain), from 6th to 7th September 2011. The scientists in charge of every research tool attended the workshop. Although a general framework was established for sampling and data collection, some adaptation was necessary depending on the local special conditions. The logistic functioning of the procedure was also tested with a pilot study, which was organized in Cadiz (children) and Madrid (adolescents), to check every step of the procedure, from sampling to data processing.

Finally, a manual of operations was ready to be followed by all participating research centers. This manual includes the whole set of data collection methodology and a detailed description of all instruments for the core study data. A protocol for data-entry includes specifications aimed at minimising coding errors. Standardised protocols for the quality control and first round of data cleaning to be applied by all participating centers were set-up. Central database architecture for centralisation of data from the different participating centres and for the different aspects of the study is being designed. A central protocol for anonymous data cross-linkage and a central analytical plan was developed.

### Ethical aspects

This project followed the ethical standards recognized by the Declaration of Helsinki (reviewed in Seoul, Republic of Korea in October 2008) and the EEC Good Clinical Practice recommendations (document 111/3976/88, July 1990), and current Spanish legislation regulating clinical and biomedical research in humans, personal data protection and bioethics (Royal Decree 561/1993 on clinical trials and 14/2007, 3rd July, for Biomedical research). The study protocols were approved by the Ethics Committee of the Hospital *Puerta de Hierro* (Madrid, Spain), the Bioethics Committee of the CSIC, and the Ethics Committe for Research Involving Human Subjects at UCA. The study was explained to the participants before starting, and the volunteers, parents or tutors signed an informed consent.

## Discussion

Results from longitudinal studies are needed to elucidate the influence of PA, sedentary behaviours, and fitness levels at childhood and adolescence on the likelihood of having disturbances in the cardiovascular disease risk factors later in life.

Studying the genetic markers related to different cardiovascular disease risk factors and their relationship to several phenotypic characteristics of the population will establish a new and better understanding of: 1) the pathogenic mechanism that are involved in non-communicable diseases, that is, a better understanding on the disease aetiology; 2) to refine the genetic and environmental effects; 3) to identify individuals at risk of the development of complications and the identification of those likely to be resistance to interventions; 4) to inform strategies of targeted disease prevention.

The findings of the UP&DOWN study may help the Health Information Systems and policy makers to identify the target population for primary prevention and health promotion policies, and to develop and test preventive strategies. Moreover, these data will allow following the trends at population level, as well as to modify/adapt/create new evidence-based PA guidelines at national level. The findings will also serve as a scientific platform for interventional studies.

## Abbreviations

ACE: Angiotensin converting enzyme; AFINOS: Physical Activity as a Preventive Measure for Overweight Obesity Infection Allergies and Cardiovascular Risk Factors in Adolescents; ALPHA: Assessing Levels of Physical Activity and Fitness; DS: Down syndrome; CSIC: Spanish National Research Council; EDTA: Ethylene diamine tetraacetic acid; EUROFIT: European Physical Fitness Test Battery; FTO: Fat mass and obesity associated; HDL-c: High density lipoprotein cholesterol; IFIS: International Fitness Scale; LDL-c: Low density lipoprotein cholesterol; PA: Physical activity; PANASN: Positive and Negative Affect Schedule for children and adolescents; PAQ-A/C: Physical Activity Questionnaire for Adolescents/Children; PCR: Polymerase chain reaction; PGC1-α: Peroxisome proliferator-activated receptor gamma coactivator 1-alpha; SHAPES: School Health Action Planning and Evaluation System; UAM: Autonomous University of Madrid; UCA: University of Cadiz; UCM: Complutense University of Madrid; YSBQ: Youth Sedentary Behaviour Questionnaire.

## Competing interests

The authors declare that they have no competing interests.

## Authors’ contributions

JCP, OLV, JRR and AM conceived the study and obtained funding. JCP, ACB, DMG and JRR drafted the manuscript. ACB, DMG and JRR contributed equally to this work. SGM, VCS, CS, AMV, FB, AGG, FGG, OLV and AM participated in the study design and critically revised the manuscript. Final approval of the version to be published was obtained from each of the authors.

## Pre-publication history

The pre-publication history for this paper can be accessed here:

http://www.biomedcentral.com/1471-2458/14/400/prepub

## The UP&DOWN Study Group

Coordinator: Ascension Marcos.

Principal Investigators: Ascension Marcos, Jose Castro-Piñero, Oscar L. Veiga and Fernando Bandres.

Scientific coordinators: David Martinez-Gomez (chair), Jonatan R. Ruiz (co-chair), Ana Carbonell-Baeza, Sonia Gomez-Martinez, and Catalina Santiago.

Spanish National Research Council: Ascension Marcos, Sonia Gomez-Martinez, Esther Nova, Ligia-Esperanza Diaz, Belen Zapatera, Ana M. Veses, Jorge R. Mujico, Aurora Hernandez and Alina Gheorghe.

University of Cadiz: Jose Castro-Piñero, Jesus Mora-Vicente, Jose L. Gonzalez-Montesinos, Julio Conde-Caveda, Jonatan R. Ruiz (University of Granada (UGR)), Francisco B. Ortega (UGR), Carmen Padilla Moledo, Ana Carbonell Baeza, Palma Chillon (UGR), Jorge del Rosario Fernandez, Ana Gonzalez Galo, Gonzalo Bellvis Guerra, Alvaro Delgado Alfonso, Fernando Parrilla, Roque Gomez, and Juan Gavala.

Autonomous University of Madrid: Oscar L. Veiga, H. Ariel Villagra, Juan del-Campo, Carlos Cordente (UPM), Mario Diaz, Carlos M. Tejero, Aitor Acha, Jose M. Moya, Alberto Sanz, David Martinez-Gomez, Veronica Cabanas-Sanchez, Gabriel Rodriguez-Romo (UPM), Rocio Izquierdo, Laura Garcia-Cervantes, and Irene Esteban-Cornejo.

Complutense University of Madrid: Fernando Bandres, Alejandro Lucia (European University of Madrid (UEM)), Catalina Santiago (UEM), and Felix Gomez-Gallego (UEM).
